# Polycistronic Expression System for *Pichia pastoris* Composed of Chitino- and Chitosanolytic Enzymes

**DOI:** 10.3389/fbioe.2021.710922

**Published:** 2021-08-18

**Authors:** Michal B. Kaczmarek, Katarzyna Struszczyk-Swita, Meng Xiao, Mirosława Szczęsna-Antczak, Tadeusz Antczak, Magdalena Gierszewska, Alexander Steinbüchel, Maurycy Daroch

**Affiliations:** ^1^ School of Environment and Energy, Peking University Shenzhen Graduate School, Shenzhen, China; ^2^ Institute of Molecular and Industrial Biotechnology, Lodz University of Technology, Lodz, Poland; ^3^ Department of Physical Chemistry and Physicochemistry of Polymers, Faculty of Chemistry, Nicolaus Copernicus University in Toruń, Toruń, Poland; ^4^ International Center for Research on Innovative Biobased Materials (ICRI-BioM), International Research Agenda, Lodz University of Technology, Lodz, Poland

**Keywords:** chitin, chitosan, enzymatic modification, polycistronic expression, self-processing 2A sequence, chitin deacetylase, chitinase, chitosanase

## Abstract

Chitin is one of the most abundant biopolymers. Due to its recalcitrant nature and insolubility in accessible solvents, it is often considered waste and not a bioresource. The products of chitin modification such as chitosan and chitooligosaccharides are highly sought, but their preparation is a challenging process, typically performed with thermochemical methods that lack specificities and generate hazardous waste. Enzymatic treatment is a promising alternative to these methods, but the preparation of multiple biocatalysts is costly. In this manuscript, we biochemically characterised chitin deacetylases of *Mucor circinelloides* IBT-83 and utilised one of them for the construction of the first eukaryotic, polycistronic expression system employing self-processing 2A sequences. The three chitin-processing enzymes; chitin deacetylase of *M. circinelloides* IBT-83, chitinase from *Thermomyces lanuginosus,* and chitosanase from *Aspergillus fumigatus* were expressed under the control of the same promoter in methylotrophic yeast *Pichia pastoris* and characterised for their synergistic action towards their respective substrates.

## Introduction

Chitin is one of the most abundant, right after cellulose and lignin, naturally occurring biopolymer being the basic component of shells of crustaceans, insects and the cell walls of filamentous fungi (Al Sagheer et al., 2009; Marei et al., 2016; Ghormade et al., 2017). These are considered as burdensome waste products of marine and biotechnology industries. According to the report of the Food and Agriculture Organization of the United Nations (FAO), the estimated annual production of farmed shrimp only for Asian countries is approx. 2.5 million tons (FAO, 2017), of which 35–45% (w/w) are waste (shells and heads), which is about 1 million tons per year (Suryawanshi et al., 2019). Currently, over 60% of biotechnology companies use fungal organisms for various purposes, including brewing, wine and bakery industries, production of antibiotics and recombinant proteins for pharmaceutical applications, production of organic acids and recombinant enzyme proteins. It is estimated that the brewing industry in Brazil, the United States and China generates approximately 2.1 million tonnes of waste yeast biomass annually (Thiago et al., 2014). The China Pharmaceutical Industry Association reported that more than 80% of total antibiotic production is penicillin produced by the Ascomycota species *Penicillium* sp. This process produces 1.2 million tonnes of bio-waste per year, mainly biomass of the above-mentioned moulds (Zhang et al., 2016). Wherein, the content of chitin in the cell wall of filamentous fungi may vary from 22 to 44% depending on the class of fungi (Synowiecki and Al-Khateeb, 1997; Chatterjee et al., 2005).

Despite interesting biological properties of chitin, its application is significantly limited primarily due to the highly crystalline structure of the polymer and the lack of solubility in commonly accessible solvents (Synowiecki and Al-Khateeb, 2003). Nowadays, products of chitin modification attract a significant interest. Their distinct physico-chemical properties, solubility in numerous media, increase the spectrum of their industrial applications. At present, N-deacetylated derivative of chitin (chitosan) and products of its degradation (chitooligosaccharides) are obtained by thermo-chemical and physical processes, which have several disadvantages (Hayes, 2012). The most important disadvantages are large amount waste that is harmful to the environment, the high energy consumption of processes and uncontrolled degradation of the polymer leading to the formation of a heterogeneous mixture of products with different physico-chemical properties, thereby affecting their biological properties (Kaczmarek et al., 2019).

Enzymatic modification of chitin and its derivatives may be a promising alternative to conventional methods. Biotransformation of chitin into chitosan through enzymatic deacetylation can be achieved with chitin deacetylases (EC 3.5.1.41). This enzymatic reaction has several advantages over the traditional chemical process, most importantly the production of chitosan with higher molecular weight and the desired degree of acetylation (Grifoll-Romero et al., 2018). Other enzymes involved in chitin and chitosan conversion are hydrolases: chitosanases (EC 3.2.1.132) and chitinases (EC 3.2.1.14). Both of them catalyse the hydrolysis of glycosidic bonds but differ in substrate specificity, the former hydrolysing bonds of chitosan and the latter of chitin (Nguyen et al., 2018). Due to the high specificities of these catalytic proteins, they are easy to control and result in products with a strictly defined chain distribution. Unfortunately, the multi-stage nature of these processes (deacetylation, depolymerisation), which are currently carried out in separated processes using disjointly operating biocatalysts, and the costs associated with obtaining each of them, make these methods economically uncompetitive.

Here we describe the synthesis of the first eukaryotic polycistronic expression system enabling the simultaneous expression of three genes encoding proteins with chitin- and chitosanolytic activity under the control of one promoter. The gene encoding chitin deacetylase from *Mucor circinelloides* IBT-83 strain was firstly heterologously expressed in yeast *Pichia pastoris* and biochemically characterised. Other genes encoding chitinase from *Thermomyces lanuginosus* (Zhang et al., 2015) and chitosanase from *Aspergillus fumigatus* (Chen et al., 2012) were synthesized and used for the preparation of a polycistronic expression vector. The functioning of the this multi-gene system is based on the viral self-processing 2A sequences that have been identified in Foot and Mouth disease Viruses (FMDV) belonging to the *Picornaviridae* family (Donnelly et al., 2001b). These short peptide sequences (approx. 20 aa) inhibits the formation of a peptide bond between C-terminal glycine and proline, causing the phenomenon of so-called ribosomal skipping, and thus spontaneous initiation of the synthesis of a new polypeptide (Donnelly et al., 2001a). The presence of several sequentially acting biocatalysts in one reaction mixture is likely to result in cascade biocatalysis under *in vitro* conditions thanks to the decrease of the diffusion path of intermediate metabolic products between individual biocatalysts (Liu et al., 2014), and could result in the significant reduction of the costs of the process.

## Materials and Methods

### Chemicals

The following reagents were purchased: fast digest restriction enzymes: *Not*I, *Sac*I and *Xba*I, Pierce BCA protein assay Kit, ATP solution (100 mM) (Thermo Scientific), Q5® High-Fidelity DNA polymerase, Thermostable *Taq* ligase, T4 polynucleotide kinase (New England Biolabs), Taq2x MasterMix (Tiangen), DNA Clean and Concentrator™-5 (Zymo Research), Wizard® SV Gel and PCR Clean-Up System and PureYield™ Plasmid Miniprep System (Promega), Zeocin (Invitrogen), chitin from shrimp (*Pandalus borealis*), chitosan (MW 190–310 kDa) (Sigma Aldrich).

### Strains Origins and Cultivation

The strain of filamentous fungi *Mucor circinelloides* IBT-83 from the culture collection of the Institute of Molecular and Industrial Biotechnology (Lodz, Poland) was used as a source of native chitin deacetylase *chda*I and *chda*II genes. Genes coding two native chitin deacetylases from *M. circinelloides* IBT-83 were isolated and molecularly cloned as described previously (Kaczmarek et al., 2016).

*E. coli* DH5α transformants were grown in LB low salt medium [1% (w/v) tryptone, 0.5% (w/v) yeast extract, 0.5% (w/v) NaCl] supplemented with 25 μg/ml Zeocin. LB agar plates [1% (w/v) tryptone, 0.5% (w/v) yeast extract, 0.5% (w/v) NaCl 1.5% (w/v) agar], supplemented with 25 μg/ml of Zeocin, were used to screen the pPICZαC bacterial transformant clones.

All culture media and ingredients used for *Pichia pastoris* experiments were prepared according to the protocol from the Easy select Pichia expression Kit (Invitrogen). Cells were grown in 250 ml baffled shake flasks covered with two layers of cotton gauze. *Pichia pastoris* KM71H strain was grown in YPD medium [1% (w/v) yeast extract, 2% (w/v) peptone and 2% (w/v) glucose]. Transformed KM71H cells were grown in buffered minimal glycerol (BMD) medium [1% (w/v) yeast extract, 2% (w/v) peptone, 1.34% (w/v) Yeast Nitrogen base YNB, 4x10^−5^% (w/v) biotin and 1% (w/v) glycerol, 200 mM potassium phosphate buffer, pH 6.0] and buffered minimal methanol (BMM) medium [1% (w/v) yeast extract, 2% (w/v) peptone, 1.34% (w/v) Yeast, Nitrogen base YNB, 4x10^−5^% (w/v) biotin and 0.5% (w/v) methanol, 200 mM potassium phosphate buffer, pH 6.0]. Transformed KM71H cells were selected on YPDS agar plates [1% (w/v) yeast extract, 2% (w/v) peptone, 2% (w/v) glucose, 2% (w/v) agar] supplemented with 100 μg/ml Zeocin.

### Substrate Preparation

#### 0.5% Chitosan in Hydrochloric Acid

The amount of 0.5 g of chitosan (MMW, DD 75–85%, viscosity 200 cP) was mixed with 95 ml of 0.1 mM HCl solution (pH 4.0) and left at room temperature overnight, with vigorous stirring. Next, concentrated HCl was added in small portions (50–100 μl) to complete polymer dissolution, which was adjusted with 0.1 mM HCl to the final volume of 100 ml (pH 4.5).

#### 2% Chitosan in Acetic Acid

The amount of 2 g of chitosan (MMW, 78.13 ± 0.14%viscosity 200 cP) was mixed with 95 ml of 2% acetic acid and left at room temperature overnight, with vigorous stirring. Next, the solution was adjusted with 2% acetic acid to the final volume of 100 ml. The substrate prepared in this way was diluted to a concentration of 0.5% just before the reaction.

#### Colloidal Chitin

Colloidal chitin was prepared according to the previously published methodology (Souza et al., 2009). The amount of 5 g of chitin (chitin from shrimp *Pandalus borealis*, Sigma Aldrich) was mixed with 60 ml of concentrated HCl and left at room temperature overnight, with vigorous stirring. Next, 200 ml of ice-cold 95% ethanol was added to the mixture and incubated overnight at 4°C with vigorous stirring. The precipitate was collected by centrifugation at 5,000 *g* for 20 min at 4°C and transferred to a glass funnel with filter paper (80 g m^−2^). Colloidal chitin was washed with sterile distilled water until the substrate became neutral (pH 7.0). Colloidal chitin was freeze-dried and stored at 4 or −20°C (long term).

### Multiple Sequence Alignment and Phylogenetic Analysis

The sequences of the catalytic domains (NodB) of the ChDaI and ChDaII proteins were included for multiple sequence alignment against other members of the CE4 deacetylase family. A multi-sequential comparison was made using Clustal Omega which use an alignment engine for aligning profile hidden Markov models (HMMs) to each other instead of the conventional dynamic programming and profile alignment (Sievers and Higgins, 2018). ChDaI and ChDaII were analysed with chitin deacetylase from *Mucor rouxii Mr*CDA (Uniprot: P50325); chitin deacetylase from *Rhizopus circinans Rc*CDA (Uniprot: A7UMZ0); chitin deacetylase from *Gongronella butleri Gb*CDA (Uniprot: Q8J2N6); chitin deacetylase from *Colletotrichum lindemuthianum Cl*CDA (PDB: 2IW0); chitin deacetylase from *Pestolotiopsis* sp. *Pes*CDA (GenBank: APH81274.1); chitin deacetylase from *Aspergillus nidulans An*CDA (PDB: 2Y8U); CE4 deacetylase isolated from the marine environment *Arthrobacter* sp. *Ar*CE4A (PDB: 5LGC); peptidoglycan deacetylase from *Streptococcus pneumoniae Sp*PgdA (PDB: *2C1G*); CE4 esterase from *Bacillus subtilis Bs*PdaA (PDB: 1W17); chitooligosaccharides deacetylase from *Vibrio parahaemolyticus Vp*COD (PDB: 3WX7); chitooligosaccharides deacetylase from *Vibrio cholerae Vc*COD (PDB: 4NY2); chitooligosaccharides deacetylase from *Shewanella woodyi Sb*COD (GenBank: ABN60929.1). Amino acid sequences of family CE4 CAZYmes (www.CAZY.org) were retrieved from Uniprot database, GeneBank and Protein Data Bank (PDB).

### Chitin Deacetylase Activity Assay

Chitin deacetylase activity was determined based on the amount of acetic acid released during the chitosan deacetylation reaction. 1 ml of 0.5% (w/v) chitosan solution and 100 µl of the supernatant obtained during proteins expression were preincubated for 2 min at 50°C, each in separate tube. The reaction was initiated by combining both the enzyme and the polymer solution and carried out for 120 min at 50°C in stirred (300 rpm) sterile 1.5 ml microcentrifuge tubes. Subsequently, the samples were incubated for 20 min at 80°C to inactivate the enzyme and cooled down. The amount of acetic acid released during the reaction was determined by GC-MS gas chromatography using Stabilwax®-DA; 30 m; 0.18 mm ID; 0.18 µm column.

### Effect of Temperature and Metal Ions on Recombinant Chitin Deacetylase

To determine the optimum temperature for recombinant chitin deacetylase from *M. circinelloides* IBT-83, enzymatic reactions were performed at various temperatures ranging from 20 to 70°C. Reactions were performed according to the methodology described above. The effect of different metal ions on enzyme activity was verified by adding metal ions: Cu^2+^, Fe^2+^, Mg^2+^, Mn^2+^, Zn^2+^, Ni^2+^ to the enzymatic reaction to a final concentration of 1 mM or 10 mM.

### Chitinase Activity Assay

Chitinase activity was determined as follows: 500 µl of 1% (w/v) colloidal chitin dissolved in citric buffer (pH 4.5) and 250 µl of the supernatant obtained during proteins expression were preincubated for 2 min at 50°C, each in separate tube. The reaction was initiated by combining both the enzyme and the polymer solution and carried out for 60 min at 50°C in stirred (550 rpm) sterile 1.5 ml microcentrifuge tube. Subsequently, the samples were incubated at 100°C for 5 min to inactivate the enzyme and cooled down. The amount of reducing sugars obtained during the reaction was determined using the Somogyi-Nelson method (Nelson, 1944).

### Chitosanase Activity Assay

To chitosanase activity was determined according to the methodology described in section above. with the exception of the substrate, which in this case was 0.5% (w/v) chitosan in 2% (v/v) acetic acid solution.

### Determination of the Degree of Deacetylation

The degree of deacetylation of products obtained by the action of recombinant chitin deacetylases was determined using the method of potentiometric titration, according to the Broussignac method (Muzzarelli et al., 1997). Precipitated and lyophilised products of the reactions were dissolved in 0.02 M hydrochloric acid to a final concentration of 0.2% (w/v). The solutions were then titrated with 0.1 M NaOH. The two endpoints (PK) of titration observed in the diagram of ΔpH = f (V), corresponding to the titrant volumes V_1_ and V_2_ defined below, were determined by the first derivative method. For this purpose, plots of the dependence ∆pH/V = f (V) were prepared, where ∆pH and ∆V denote the increments between successive changes in pH and the volume of the titration reagent. The deacetylation degree was calculated from the following formula:

$$DD=\frac{M_{GlcNAc}\cdot c_{NaOH}\cdot \left(V_{2}-V_{1}\right)}{m_{Ch}+c_{NaOH}\cdot \left(M_{GlcNAc}-M_{GlcN}\right)\cdot \left(V_{2}-V_{1}\right)}\cdot 100\%$$ (1)

where: DD—degree of deacetylation (%), $c_{NaOH}$—titrant concentration (mol∙dm^−3^),$m_{Ch}$—chitosan sample mass (g), $V_{1}$—the volume of titrant used to neutralise hydrochloric acid (dm^3^), $V_{2}$—the volume of titrant used to neutralise the sum of hydrochloric acid and protonated amine groups (dm^3^), $M_{GlcNAc}$—molar mass of: 2-acetamido-2-deoxy-D-glucopyranose (g∙mol^−1^), $M_{GlcN}$—molar mass of: 2-amino-2-deoxy-D-glucopyranose (g∙mol^−1^).

### Chitin Deacetylases Expression Vectors Preparation

The *chda*I and *chda*II (without native signal peptide) genes used for heterologous expression in *P. pastoris* were amplified from previously described pJET transformants (Kaczmarek et al., 2016). The selected restriction sites: *Not*I and *Xba*I were incorporated into the PCR products by using specially designed primers (chdaI_For 5′-ATT​TGCG​GCC​GCTGA​CAC​CTC​CGC​AAA​TTA​CTG​G-3′ chdaI_Rev 5′-TGCTCT​AGAGC​AAG​TAA​CAA​GGT​AGC​AAT​AAA​GGC​AG-3′, chdaII_For 5′-ATT​TGCGG​CCG​CTGC​TAC​TTC​CAC​CAA​ATC​CGC-3′, chdaII_Rev 5′- TGCTCT​AGAGCG​AAA​ATG​TAA​GCA​GCA​ACG​GC-3′). The resultant PCR products were cloned into the *P. pastoris* expression vector pPICZαC (Invitrogen) using commercially available chemocompetent cells DH5α (TianGen). The Zeocin resistant *E. coli* pPICZ_chdaI and pPICZ_chdaII transformants were selected by culturing on LB agar plates, supplemented with 25 μg/ml of Zeocin at 37°C.

### Polycistronic Vector Synthesis

#### Generation of Plasmid for Polycistronic Expression

**FIGURE 1:**
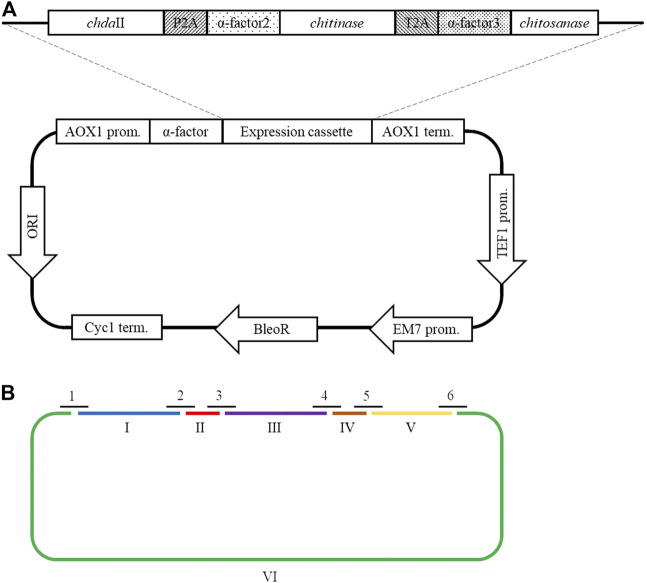
**(A)** The map of the pCHIT expression vector and a fragment of the expression cassette; **(B)** Simplified diagram of the synthesis of a polycistronic vector pCHIT by use of LCR. 1-6 bridge sequences - black lines (Supplementary Table S2); I-VI - vector components - colored lines (Supplementary Table S1).

A polycistronic expression vector was prepared based on the commercial vector pPICZαC. The *chda*II gene was amplified from previously described pJET transformants (Kaczmarek et al., 2016) using phosphorylated chdaII_FLCR 5′-GCT​ACT​TCC​ACC​AAA​TCC​GCC-3′ and chdaII_RLCR_5′-GAAAATGTAAGCAGCAACGGCA-3′ primers. The gene coding chitinase from *Thermomyces lanuginosus* (Zhang et al., 2015) was enriched with the sequence coding the α-factor secretion signal, codon optimised and synthetised by Genewiz (Suzhou, China). The same approach was used for the gene coding chitosanase from *Aspergillus fumigatus* (Chen et al., 2012). The sequences encoding the α-factor secretory sequences preceding both genes were optimised considering the degeneracy of the genetic code, limiting the occurrence of spontaneous homologous recombination. To generate 2A self-processing sequences, the ssDNA oligos were synthetised based on the P2A (ATNFSLLKQAGDVEENPGP) and T2A (EGRGSLLTCGDVEENPGP) amino acid sequences and hybridised according to the program 95°C—2min; 70°C–10 min; 72°C–5 min; 4°C–the end. The synthesis of the polycistronic vector was carried out using the LCR method (Kok et al., 2014). The above-described sequences used to synthesise the vector were amplified using phosphorylated primer sequences. Bridging oligonucleotides complementary to the ends of neighbouring DNA parts (half-bridging oligos) were designed with a target melting temperature (Tm). Figure 1A shows the map of the expression vector and a fragment of the expression cassette. The polycistronic vector pCHIT was synthesised from six DNA fragments, the size of which ranged from 50 bp to 3,500 bp. Individual fragments were amplified using the phosphorylated primers listed in the Supplementary Table S1.

#### Ligase Cycled Reaction–LCR

The LCR reaction was performed based on the modified method of Roth et al. Roth et al, (2014) as previously described by as (Liang et al., 2019). DNA fragments for a specific cycled ligation assembly reaction were mixed and diluted with ultra-pure H_2_O to obtain a mixture with a concentration of 20 nM for each fragment. LCR assembly utilises single-stranded bridging oligos complementary to the ends of DNA parts to be assembled. The melting temperature of designed (half)bridging oligos was around 58–62°C. Bridging oligonucleotides (Supplementary Table S2) were diluted with ultra-pure H_2_O to a final concentration of 200 µM. Thermostable *Taq* ligase (NEB) was used for the assembly reaction. The composition of the final reaction mixture (20 µL) is as follows: 2 nM for each DNA fragment, 10 nM bridging oligonucleotides, 1xTaq ligase buffer, 5% DMSO, and 40 U Taq ligase. The reaction was cycled according to the following program: initial denaturation 95°C (2 min) followed by 30 cycles of 95°C (30 s), 60°C (2 min), final incubation performed at 55°C (10 min), and samples stored at 4°C until transformed to *E. coli* DH5α (Tiangen, Beijing, China) according to the manufacturer’s protocol. Figure 1B shows a simplified scheme for the synthesis of the polycistronic vector pCHIT using the LCR method.

### Transformation of *Pichia pastoris* KM71H Strains and Selection of Positive Transformants

Plasmid DNA containing one of the genes of interest was linearised by overnight restriction digestion using *Sac*I (Thermo Scientific). Purified, linearised plasmid (∼100 ng) was added into the electrocompetent KM71H cells. The prepared mixture was carefully transferred into the slide of the chilled and sterile cuvette. After electroporation (Voltage 1.5 kV, Resistance 125 ohms, Pulse length 3 msec.), cells were immediately resuspended in 500 µl of 1 M cold sorbitol. Then 500 µl of YPD pH 7.5 were added into the mixture. Cells were incubated with horizontal shaking at 30°C for at least 3–4 h. After transformation, cells were pelleted, 700 µl of supernatant were removed, cells were resuspended in the remaining media and plated on YPDS agar plates supplemented with 100 μg/ml Zeocine and incubated for 2 days at 30°C.

Colony PCR was used to confirm the presence of the expression cassette in the genome of *Pichia pastoris* transformants. Single colonies were replated on fresh YPD agar plates supplemented with 100 μg/ml Zeocine and resuspended in 10 µl sterilee water, heated to 95°C for 5 min, centrifuged at top speed and used as a template for PCR reaction containing Taq2x DNA MasterMix (Tiangen, Beijing, China), and universal primers (AOX1_For5′-GACTGGTTCCAATTGACAAGC-3′, AOX1_Rev 5′-GCA​AAT​GGC​ATT​CTG​ACA​TCC-3′).

### *Pichia pastoris* Cultivation Conditions

A single colony from fresh YPD agar plates supplemented with 100 μg/ml Zeocine was used to inoculate 25 ml BMGY medium in a 250 ml baffled flask and grew at 28–30°C in a shaking incubator (250–300 rpm) for 18 h. The preculture with OD_600_ ranging from 10 to 12 was used to inoculate 100 ml of BMGY medium in 1,000 ml baffled flask to final OD_600_ 0.2 and grew until the culture reaches OD_600_ ∼2–3. The grown cells were harvested at 1.500–3.000×*g* for 5 min at room temperature and next resuspended in 20 ml of BMMY medium (100 ml baffled flasks covered with two layers of sterile gauze or cheesecloth). Every 24 h, pure methanol was added to the culture to the final concentration of 0.5%. The samples from 0, 24, 48, 72 and 96 h of culture were taken to measure protein concentration, prepare SDS-PAGE analysis and check the enzymatic activity of recombinant proteins.

## Results and Discussion

### Chitin Deacetylases Multiple Sequence Alignment

**FIGURE 2:**
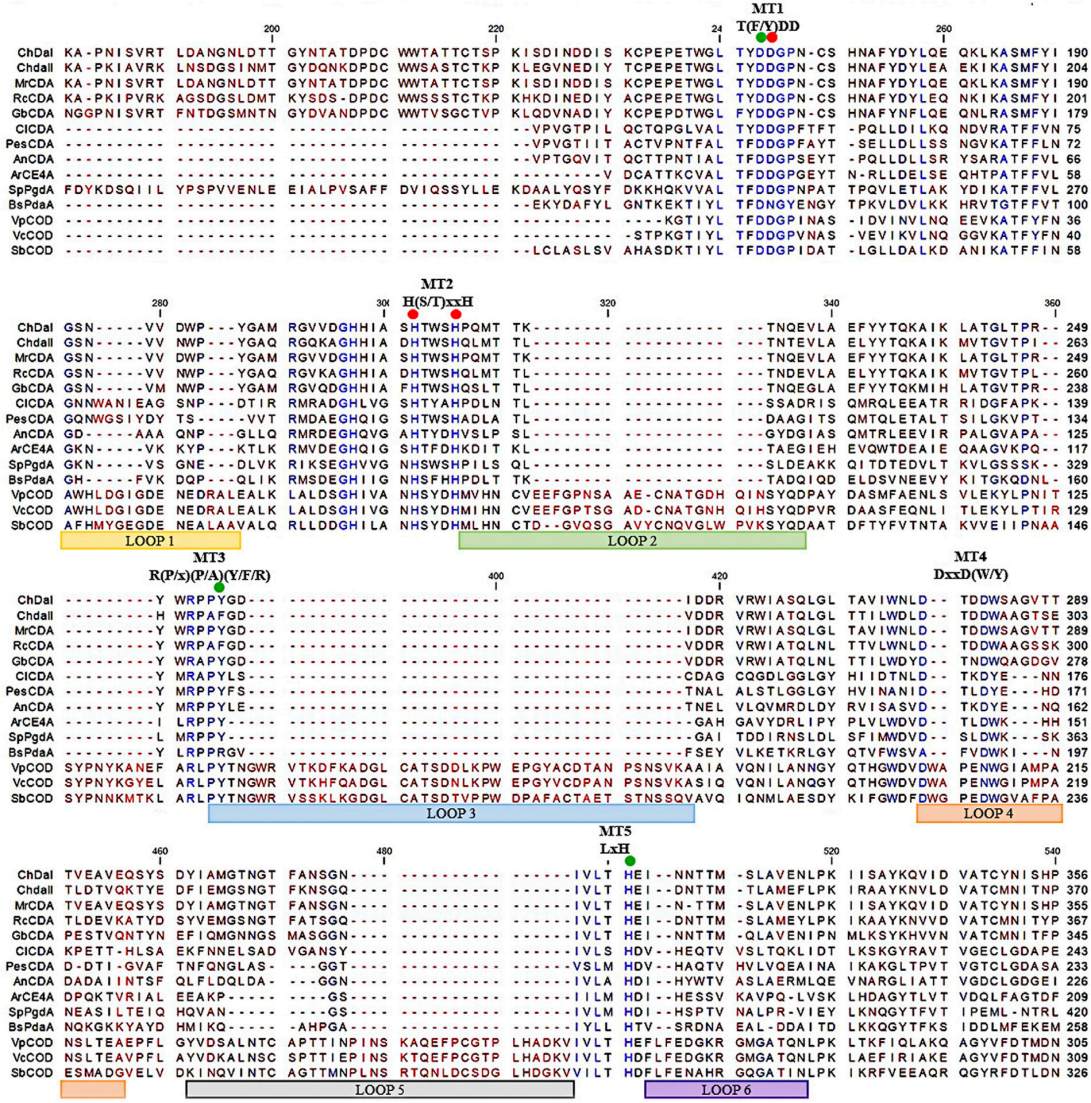
Multiple sequence alignment of chitin deacetylases ChDaI and ChDaII from *Mucor circinelloides* IBT-83. Abbreviations: chitin deacetylases from: *Mucor rouxii* MrCDA (Uniprot: P50325), *Rhizopus circinans* RcCDA (Uniprot: A7UMZ0), *Gongronella butleri* GbCDA (Uniprot: Q8J2N6), *Colletotrichum lindemuthianum* ClCDA (PDB: 2IW0), *Pestolotiopsis* sp. PesCDA (GenBank: APH81274.1), *Aspergillus nidulans* AnCDA (PDB: 2Y8U); CE4 deacetylase from marine environment *Arthrobacter* sp. ArCE4A (PDB: 5LGC); peptidoglycan deacetylase from *Streptococcus pneumoniae* SpPgdA (PDB: 2C1G); CE4 esterase from *Bacillus subtilis* BsPdaA (PDB: 1W17); chitooligosaccharides deacetylases from *Vibrio parahaemolyticus* VpCOD (PDB: 3WX7), *Vibrio cholerae* VcCOD (PDB: 4NY2), *Shewanella woodyi* SbCOD (GenBank: ABN60929.1).

Previous research has shown that the strain *Mucor circinelloides* IBT-83 is an efficient producer of intracellular chitin deacetylase (Kaczmarek et al., 2016). However, the large amount of microbial lipids produced by this strain significantly limited the efficiency of proteins purification and their enzymatic activity (Szczesna-Antczak et al., 2006). Therefore, two genes potentially encoding chitin deacetylases *chda*I and *chda*II were identified by bioinformatic analysis of the genome sequence of the filamentous fungi *Mucor circinelloides* CBS 277.49 (Project ID: 403122, Joint Genome Institute) using Blast-2.2.30 software. The *chda*I and *chda*II genes amplified on the cDNA from *Mucor circinelloides* IBT-83 cDNA, cloned and sequenced (Kaczmarek et al., 2016). The multi-sequence comparison of *in silico* translated ChDaI and ChDaII sequences against other members of the CE4 deacetylase family showed high homology in the area of the NodB domain, also called the catalytic domain. The obtained results presented in Figure 2 indicate the presence of five catalytic motifs MT1-5 crucial for the functioning of enzymes belonging to the CE4 esterase family: Motif1 (MT1) corresponding to the sequence (M/Y)DD; Motif2 (MT2) H(S/T)xxH, Motif3 (MT3) R (P/x) (P/A) (Y/F/R), Motif4 (MT4) DxxD (W/Y) and the Motif5 (MT5) including the residues LxH. The Tyr255(ChDaI)/Phe269(ChDaII) (MT3) together with Asp164(ChDaI)/Asp178(ChDaII) (MT1) and His320(ChDaI)/His334(ChDaII) (MT5) residues are directly involved in the amide bond hydrolysis reaction (Nakamura et al., 2017; Tuveng et al., 2017). Moreover, highly conserved residues Asp165(ChDaI)/Asp179(ChDaII) (MT1), His214(ChDaI)/His228(ChDaII) (MT2) and His218(ChDaI)/His232(ChDaII) (MT2) form the so-called an metal-binding triad that coordinate the metal atoms; typically one of Zn^2+^, Co^2+^, Ca^2+^; which are co-factors of chitin deacetylases (Zhu et al., 2019). Moreover, the performed multi-sequence comparison allowed for the identification of six loops in the amino acid sequences of ChDaI and ChDaII proteins. Crystallographic studies carried out previously (Andrés et al., 2014) showed that these loops are located in close proximity to the active centre of the enzyme and are involved in “capturing” the substrates subjected to deacetylation reactions.

## Expression and Enzymatic Activity of Chitin Deacetylases ChDaI and ChDaII Form *M. circinelloides* IBT-83

**FIGURE 3:**
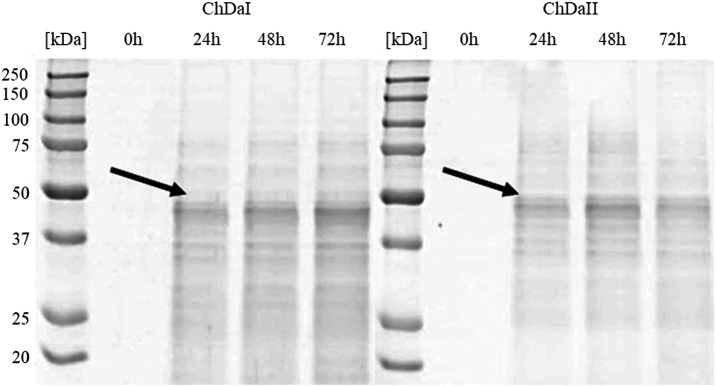
Results of electrophoretic analysis (SDS-PAGE) of proteins present in the culture medium obtained after the cultivation of selected transformants of the KM71H_pPICZ_chdaI (ChDaI) strain and the KM71H_pPICZ_chdaII (ChDaII) strain. The two expressed proteins of the molecular weight of approximately 44 kDa are indicated with arrows. The culture liquids analysed were collected at different culture times: 0, 24, 48 and 72 h from the start of methanol induction. Precision Plus Protein Unstained Standards (Bio-Rad, United States) was used as the protein size standard. 4% stacking gel; resolving gel 12%, voltage 150 V.

The multi-sequence comparison of the amino acid sequences of the ChDaI and ChDaII proteins suggest that the genes *chda*I and *chda*II identified in the filamentous fungi *M. circinelloides* IBT-83 encode these enzymes exhibiting chitin deacetylase activity. The fungal chitin deacetylases known so far are glycoproteins, for which the post-translational attachment of sugar residues, mainly mannose, is crucial for the proper functioning of enzymes. Chitin deacetylase from *Mucor rouxii* has been identified as a monomeric protein with a high content of linked mannose residues (Martinou et al., 1993). Another example of a highly glycosylated chitin deacetylase is an enzyme isolated from soil fungi of the genus *Mortierella* sp. Importantly, the enzymatic deglycosylation performed at this glycoprotein resulted in a complete loss of catalytic activity (Zhao et al., 2011). Our attempts to functionally express the *M. circinelloides* IBT-83 *chda* genes in *E. coli* were also unsuccessful (results not shown). This is most likely due to inability of this prokaryotic host to carry out the necessary post-translonational modifications. Analysis of the amino acid sequences of the ChDaI and ChDaII proteins using bioinformatic tools NetNGlyc 1.0 (http://www.cbs.dtu.dk/services/NetNGlyc/) and NetOGlyc 4.0 (http://www.cbs.dtu.dk/services/NetOGlyc/) showed that both proteins have numerous sites of potential N- and O-glycosylation. Due to the ability to carry out post-translational modifications and the simplicity of genetic manipulation, the yeast *Pichia pastoris* was selected as an efficient host organism for heterologous gene expression. The open reading frames ORF (without native signal sequences, 21 aa.) encoding ChDaI and ChDaII were successfully cloned and transformed into *Pichia pastoris* KM71H for and targeted for extracellular expression. The theoretical mass of recombinant proteins enriched with the C-terminal 6xHis tag and *Myc* Tag was 50.8 and 51.7 kDa for ChDaI and ChDaII, respectively. Figure 3 shows the results of electrophoretic analysis (SDS-PAGE) of proteins present in the culture medium obtained after culturing selected KM71H_pPICZ_chdaI and KM71H_pPICZ_chdaII transformants expressing proteins, ChDaI and ChDaII, respectively. Electrophoretic analysis showed that the mass of deacetylase I—ChDaI is approx. 48 kDa, while the mass of the second expressed protein - ChDaII is approx. 49 kDa. It is surprising that the actual molecular weight of the obtained recombinant chitin deacetylases from *M. circinelloides* IBT-83 are smaller than the determined theoretical weight of the proteins. However, it should be remembered that the migration of proteins in the SDS environment, in addition to size, may also be dependent on the structural properties of proteins themselves, their charge, and the ability to bind SDS while forming micelles. The prior research showed that for membrane proteins, the difference between the theoretical mass and the experimental mass determined during SDS-PAGE analysis could be as high as 48% (Rath et al., 2009). Moreover, *P. pastoris* yeast produces numerous extracellular proteolytic enzymes responsible for the hydrolysis of peptide bonds from the N-terminal end of proteins (Sinha et al., 2005). However, peptide mapping analysis performed on proteins extracted from the gel bands indicated that the recombinant proteins were chitin deacetylases.

**FIGURE 4:**
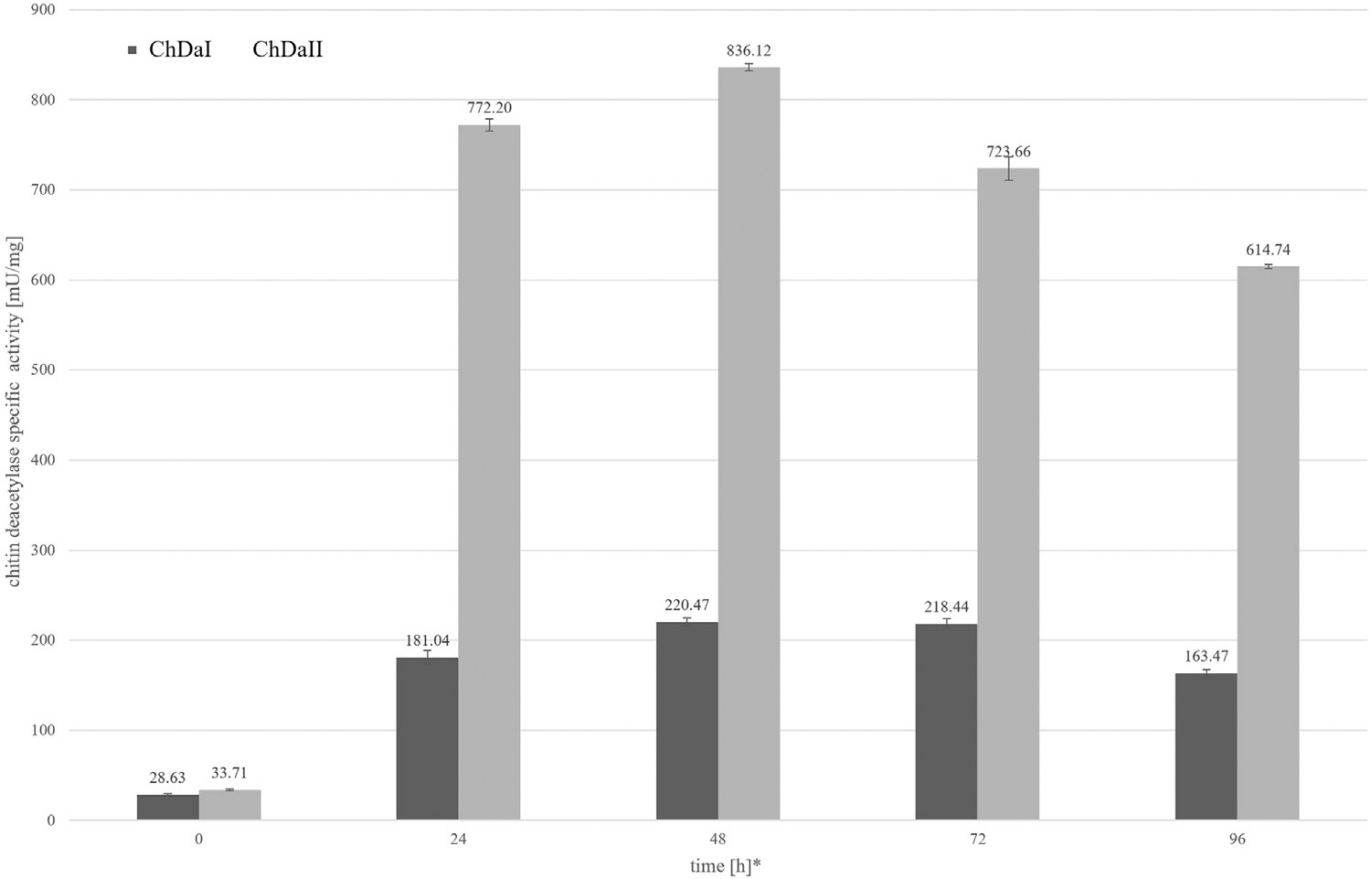
Relationship between the specific activity of chitin deacetylases and the time of cultivation. The enzymatic preparations were made from the culture medium obtained after the cultivation of transformants exhibiting the highest chitin deacetylases activity. * Cultivation time measured from the start of methanol-induced expression of ChDaI and ChDaII.

The analysis of the enzymatic activity of chitin deacetylases, carried out for the culture medium from different time points of cultivation, showed that deacetylase II has 74% higher maximum specific activity compared to deacetylase I. Figure 4 shows the dependence of the specific activity of ChDaI and ChDaII on the duration of the methanol-induced culture. Both enzymes showed the highest specific activity after 48 h of cultivation, which was 220.47 mU mg^−1^ of protein and 836.12 mU mg^−1^ for ChDaI and ChDaII, respectively. The specific activity of chitin deacetylase II adjusted for the protein concentration determined on the basis of the band intensity corresponding to the recombinant protein (Figure 3) was 3.57 U mg^−1^ (Supplementary Figure S4). The analysis of the products obtained as a result of the ChDaII activity, showed a significant increase in the degree of polymer deacetylation (DD). The average DD of the products was 96.0 ± 1.6% in relation to the substrate, the degree of deacetylation of which was DD 78.13 ± 0.14%. Since deacetylase II showed a much higher catalytic activity compared to deacetylase I, only isoform II of chitin deacetylase from *Mucor circinelloides* IBT-83 was subjected to further analyses.

Analysis of the biochemical properties of the recombinant chitin deacetylase from *Mucor circinelloides* IBT-83 allowed the determination of the optimal temperature for the action of the recombinant enzyme. The highest activity of ChDaII was determined for the reaction temperature of 50°C. Exceeding this value results in a drastic decrease in activity by approx. 90% for the temperature of 60°C and almost complete loss of ChDaII catalytic activity at 70°C (Table 1). Most fungal chitin deacetylases studied so far show the highest catalytic activity at 50°C. However, chitin deacetylases with distinct properties are also known. An example is chitin deacetylase from *Rhizopus circinans*, for which the optimal temperature was 37°C (Gauthier et al., 2008). On the other hand, native deacetylase from *C. lindemuthianum* showed higher thermostability and the highest activity at 60°C (Tokuyasu et al., 1996).

**TABLE 1:** Effect of reaction temperature on the activity of recombinant chitin deacetylase ChDaII from *Mucor circinelloides* IBT-83. The culture medium obtained after 48 h of cultivation of the selected transformant was used as the enzyme preparation.

Reaction temperature (°C)	ChDaII activity (mU ml^−1^)
**20**	34.6
**30**	171.3
**40**	212.7
**50**	288.5
**60**	29.8
**70**	1.9

Chitin deacetylases, including those of fungal origin, belong to the group of metalloenzymes. Divalent metal ions act as enzyme cofactors that are coordinated by side groups of amino acids belonging to the metal-binding triad (Figure 2). Table 2 shows the results of the percentage change in the enzymatic activity of recombinant chitin deacetylase II from *M. circinelloides* IBT-83 for reactions carried out in a medium enriched with divalent metal ions at a concentration of 1 mM or 10 mM, compared to the control for which the reaction medium was not enriched with metal ions. The obtained results clearly indicate a high dependence of the recombinant enzyme activity on the type and concentration of bivalent ions. The use of Cu^2+^ and Ni^2+^ ions significantly inhibited the activity of the tested enzyme by 44–94%. For Fe^2+^ ions, a decrease in activity was observed only at the concentration of metal ions at 10 mM. The enzyme activity increased in the presence of Mg^2+^ ions at concentrations of 1 and 10 mM and Mn^2+^ ions at a concentration of 1 mM. Interesting results were observed using Zn^2+^ ions as the enzyme cofactor. For the concentration of 1 mM, an increase in chitin deacetylase activity as much as 35% was observed. Increasing the concentration of metal ions to 10 mM resulted in a decrease in activity by 21%. Literature data show that the effect of individual metal ions on the activity of chitin deacetylases isolated from various microorganisms, mainly fungi, vary from protein to protein and it is difficult to determine the direct relationship between the amino acid sequence of proteins and their response to the presence of individual metal ions in the reaction environment. The closely related, native chitin deacetylase from *M. rouxii* (*Mr*CDA, Figure 2) showed increased activity after incubation in a buffer enriched with Zn^2+^ ions (1 mM), while the use of Mn^2+^ ions inhibited the biocatalyst (Kołodziejska et al., 1999). On the other hand, both *C. lindemuthianum* chitin deacetylase (*Cl*CDA) and ChDaII were significantly inhibited by Ni^2+^ ions despite significant sequence differences within the catalytic motifs. Furthermore, Mn^2+^ ions at a concentration of 10 mM limited only the activity of native *Cl*CDA (Tokuyasu et al., 1996). Moreover, native and recombinant enzymes derived from the same microorganisms may also show different effects of metal ions on their activity. The previous studies showed that the activity of the native chitin deacetylase from *Rhizopus circinans* is increased in the presence of Mn^2+^ and Mg^2+^ ions (1 mM) and was strongly inhibited by Cu^2+^ ions (Gauthier et al., 2008). The recombinant enzyme showed no significant changes in activity due to the presence of manganese and magnesium ions, however, like the native protein, it was strongly inhibited by Cu^2+^ ions. Chitin deacetylase from *C. lindemuthianum* expressed in *P. pastoris* also showed different properties than the native enzyme (Blair et al., 2006).

**TABLE 2:** Percentage change in enzymatic activity of recombinant chitin deacetylase ChDaII from *Mucor circinelloides* IBT-83. The culture medium obtained after 48 h of cultivation of the selected transformant was used as the enzyme preparation.

Metal ions	Percentage change in enzymatic activity (%)	
	1 mM	10 mM
Control	100	100
Cu^2+^	56	11
Fe^2+^	98	80
Mg^2+^	119	112
Mn^2+^	109	98
Zn^2+^	135	79
Ni^2+^	12	6

### Polycistronic Expression Vector Synthesis

**FIGURE 5:**
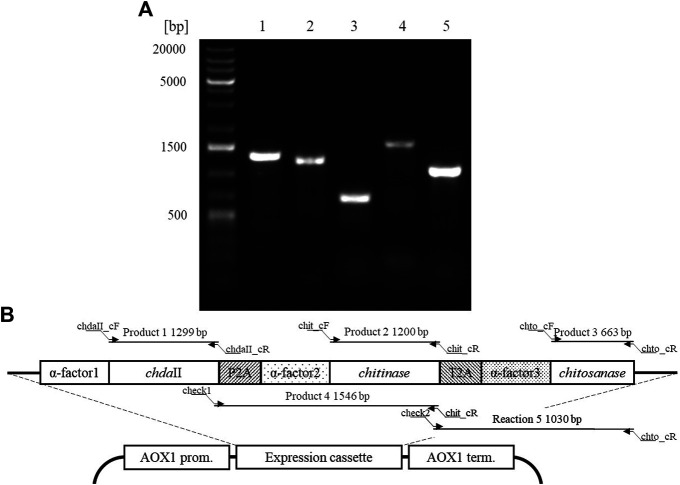
A. Electrophoretic analysis of products obtained by Colony PCR analysis (SupplementaryTable S3) carried out for a selected transformant *P. pastoris* KM71H_pCHIT. Map of the expression cassette of the polycistronic vector pCHIT with marked sizes of products obtained as a result of PCR colony analyses.

The factor limiting the use of enzymatic methods in large-scale modification of chitin substrates is the high crystalline structure of the polymer. Crystallinity limits the availability of reactive groups present in the polymer chains for enzyme action. This phenomenon is often observed in case of chitin deacetylases (Pareek et al., 2013). Moreover, the bioconversion of chitin substrates to chitooligosaccharides with the desired chain arrangement requires the action of several enzyme preparations, which increases costs of these processes. The currently used conventional thermo-chemical and chemical methods have many disadvantages, with uncontrolled degradation of polymers as the most important one. The latter results in a mixture of heterogeneous products (Struszczyk, 2000). Nevertheless, high efficiency and low costs mean that they are still used in industrial processes. It is expected that simultaneous expression of three genes encoding proteins with chitin- and chitosanolytic activity under the control of one promoter might increase the efficiency of enzymatic processes and reduce the costs associated with the production of biocatalysts. A polycistronic expression vector incorporating the genes of chitin deacetylase ChDaII, chitinase from *Thermomyces lanuginosus*, and chitosanases from *Aspergillus fumigatus* was prepared employing the LCR method. Ligase cycled reaction is an efficient and powerful tool to assemble many DNA fragments in a single reaction. The selections of genes encoding chitinase and chitosanase were based on the results of biochemical analysis of recombinant chitin deacetylase ChDaII from *Mucor circinelloides* IBT-83. Analysis of the literature data showed that the best candidate for heterologous expression of chitinase was chitinase1 (Chit1) from *Thermomyces lanuginosus*, which was already heterologously expressed in *P. pastoris* GS115 strain. The optimal temperature for this enzyme was 50°C, while the optimal pH of the reaction medium was pH 5.0 (Zhang et al., 2015). The advantage of this biocatalyst, compared to the other candidates, was that it exhibits satisfactory exo- and endochitinolytic activity against native chitin derived from powdered shrimp shells. The selected chitosanase from *Aspergillus fumigatus* was also successfully heterologously compressed in *P. pastoris* GS115. The recombinant enzyme showed high activity over a wide pH range from 4 to 7. The optimal temperature of its activity was 60°C, however, at 50°C it showed catalytic activity exceeding 80% of the maximum activity. Like native ChDaII, the chitosanase from *A. fumigatus* contained an N-terminal signal sequence of 17 amino acids (Chen et al., 2012). To avoid potential problems with the presence of the native signal peptide, a gene lacking this sequence was used for the preparation of the polycistronic vector pCHIT. The genes coding each enzyme were separated by two different A2 self-processing sequences. Verification of the assembly was performed with a series of PCR reactions using appropriate primers (Supplementary Table S3). Figure 5A shows the results of the electrophoretic analysis of PCR reaction products. The efficiency of assembling six DNA fragments, the size of which ranged from 50 bp to 3,500 bp, was 50%. Prior research showed that the LCR method allows for the assembly of eight fragments with efficiency reaching even 90%. (Roth et al., 2014). For each of the three enzymes α-factor secretion signal was used to guide the secretion of recombinant proteins. This approach was used to ensure a similar efficiency of expression and secretion of individual proteins. The degeneracy of the genetic code was used in the optimisation of secretory sequences to reduce the risk of homologous recombination between the enzymes and subsequent loss of function. Despite these efforts, these sequences showed a relatively high homology, which could have exerted an impact on the correct synthesis of the pCHIT vector. Similar sequence homology problems were observed for the viral A2 self-processing sequences. These sequences show little interspecies variability, which can also impede the correct synthesis of the polycistronic vector pCHIT. In the light of these sequence homology challenges, the efficiency of the process reaching 50% can be considered a success. De Kok et al. (2014) proved that a similar efficiency of DNA fragment assembly could be obtained only with the use of yeast recombination mechanisms. However, LCR assembly provides a much faster and easier workflow than recombination *in vivo*.

### Expression of Three Enzymes From the Polycistronic Vector

**FIGURE 6:**
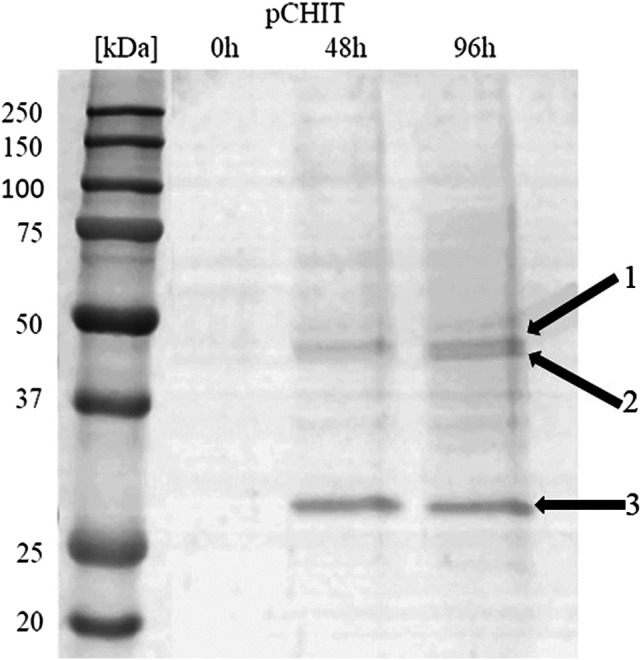
Electrophoretic analysis (SDS-PAGE) of proteins present in the after-culture medium obtained after culturing selected KM71H_pCHIT transformant. Arrows indicate following expressed proteins: 1—recombinant chitin deacetylase ChDaII from *Mucor circinelloides* IBT-83 enriched with an 18 amino acid fragment of P2A sequence M_w_∼48 kDa; 2—recombinant chitinase from *T. lanuginosus* enriched with a 17 amino acid fragment of T2A sequence M_w_∼46 kDa; recombinant chitosanase from *A. fumigatus* M_w_∼27 kDa. The culture liquids analysed were collected at different culture times: 0, 24, 48 and 72 h from the start of methanol induction. Precision Plus Protein Unstained Standards (Bio-Rad, United States) was used as the protein size standard. 4% concentrating gel; developing gel 12%, voltage 150 V.

The correctly synthesised polycistronic expression vector was linearised with *Sac*I and electroporated into *Pichia pastoris* KM71H cells. Selected transformants, for which integration of the expression vector into the host genome was confirmed by colony PCR, were subjected to methanol-induced heterologous expression. Figure 6 shows the results of electrophoretic analysis (SDS-PAGE) of proteins present in the culture medium obtained after culturing the selected KM71H_pCHIT transformant. The gels show three biosynthetic products, the mass of which ranged from 46 to 48 kDa for product 1 and 2 (chitin deacetylase ChDaII from *Mucor circinelloides* IBT-83 and chitinase from *T. lanuginosus,* both enriched with their A2 self-processing sequences, respectively), and about 27 kDa for product 3 (chitosanase from *A. fumigatus*). According to the mechanism of functioning of A2 self-processing sequences, the gene preceding the viral sequence will be enriched with the N-terminal fragment of these sequences. Therefore, the theoretical mass of chitin deacetylase II from *M. circinelloides* IBT-83 enriched with an 18 amino acid fragment of P2A sequence was 49 kDa, while the predicted mass of recombinant chitinase from *T. lanuginosus* with a 17 amino acid N-terminal fragment of the T2A sequence was 45.9 kDa. The obtained products 1 and 2 (Figure 6) correspond to ChDaII + P2A chitin deacetylase and *T. lanuginosus* chitinase enriched with T2A sequences. As it was mentioned, chitinase from *T. lanuginosus* was already expressed in *P. pastoris* GS115 strain, and its mass was 44.1 kDa. However, this variant was not enriched with the T2A viral sequence (Zhang et al., 2015). The weight of the third product (27 kDa, Figure 6) differs from the theoretical weight of chitosanase from *A. fumigatus*, which was 23.6 kDa. The results earlier showed that the recombinant chitosanase from *A. fumigatus* has a mass of approx. 28 kDa when expressed in *P. pastoris* (Chen et al., 2012). Most likely, it proves a relatively high protein glycosylation (approx. 18.6% in relation to the protein mass). Bioinformatic analysis showed that this enzyme has several potential O-linked glycosylation sites and one potential N-linked glycosylation site. Moreover, it may be related to the process of cleavage of the secretory sequence by the native KEX2 proteinase produced by the yeast *P. pastoris* (Kjeldsen, 2000). The theoretical mass of chitosanase, taking into account the secretory sequence, is approx. 32 kDa.

Recombinant proteins extracted from the two electrophoretic bands were subjected to peptide mapping analysis. Trypsin digestion was performed with proteins extracted from the upper band potentially containing *M. circinelloides* IBT-83 chitin deacetylase and *T. lanuginosus* chitinase and the lower band containing *A. fumigatus* chitosanase. The obtained peptides (from the upper band) were aligned with the *T. lanuginosus* chitinase sequence (GenBank accession number AIW06013.1) and the *M. circinelloides* f. *lusitanicus* CBS 277.49 chitin deacetylase sequence (GenBank accession number OAD01792.1). Protein sequence coverage was 62 and 6%, respectively, for recombinant chitinase from *T. lanuginosus* (Supplementary Figure S1) and ChDaII from *M. circinelloides* IBT-83 (Supplementary Figure S2). Peptides obtained after digestion of the lower band protein were aligned with the endochitinase sequence from *Asperillus* sp. CJ22-326 (GenBank accession number ABZ88800.1) with the protein sequence coverage at 39% (Supplementary Figure S3). The obtained results clearly indicated the presence of recombinant proteins in the culture medium confirming the correct functioning of the A2 self-processing viral sequences.

Viral self-processing A2 sequences have been successfully used for multi-gene polycistronic expression in cells of various eukaryotes, incl. human and mouse cell lines, plants and yeast cells (Geier et al., 2015). The advantage of using A2 sequences for multi-gene expression is their small size compared to IRES (*Internal Ribosome Entry Sites*) sequences, which significantly reduces the size of multi-gene constructs, and thus facilitates their transformation into efficient producer organisms. Early studies showed that the amount of proteins obtained from the expression of genes upstream and downstream of A2 sequences is the same or very similar (Halpin et al., 1999). This was supposed to be another advantage over IRES sequences. Subsequent *in vitro* analysis of the translation process showed some disproportions in the amount of proteins expressed using the A2 sequence. De Felipe et al. (De Felipe et al., 2006) showed that proteins encoded ahead of A2 sequences are produced in a slightly higher amount than proteins that genes are located behind them. These differences, however, were not as significant as when the IRES sequence was used. Our results may indicate that the expression efficiency of such polycistronic systems also depends on the size of the expressed genes. The band indicating the presence of chitosanase, the smallest and the third of the three co-expressed genes, appeared in the culture medium in the highest amounts (Figure 6). It is unclear at this stage as to whether this difference is due to translation, folding or protein export.

## Enzymatic Activity of the Multi-Enzyme Cocktail

The culture medium obtained after 96 h of methanol-induced cultivation of *P. pastoris* KM71H_pCHIT strain was analysed for the activity of chitin deacetylase, chitinase and chitosanase Table 3). The activity of recombinant chitin deacetylase was 372 mU mg^−1^ of protein. However, taking into account the intensity of the band peptide identified as chitin deacetylase II shown in Figure 6, the adjusted specific activity of the recombinant enzyme was 3.97 U mg^−1^ (Table 3; Supplementary Figure S4). The obtained results indicate that the specific activity of the enzyme expressed in the multigene system is slightly higher than that of ChDaII after expression of the monogenic vector. This may indicate the synergistic action of biocatalysts in the multi-enzyme cocktail. Pre-hydrolysis of the glycosidic bonds loosens the dense structure of the chitin substrate, thereby increasing the availability of acetyl residues for the action of chitin deacetylase. The degree of deacetylation of the products obtained by the action of ChDaII chitin deacetylase from the multigene system reached 85.22 ± 0.17%.

**TABLE 3:** Enzymatic activity determined in the post-culture liquid obtained after expression of the pCHIT transformants.

Enzyme	Substrate	Protein concentration (mg ml^−1^)	Activity (U ml^−1^)	Specific activity (U mg^−1^)	Adjusted protein concentration (mg ml^−1^)^a^	Adjusted specific activity (U mg^−1^)^a^
Chitin deacetylase	0.5% chitosan^b^	0.68	0.25	0.37	0.063	3.97
Chitinase	Colloidal chitin		1.99	2.93	0.039	51.03
Chitosanase	0.5% chitosan^c^		3.46	5.09	0.295	11.73

a Determined on the basis of the intensity of the band corresponding to the recombinant protein.

b Dissolved in hydrochloric acid.

c Dissolved in 2% acetic acid.

The activities of recombinant chitinase and chitosanase were determined by measuring the concentration of reducing sugars. Since both enzymes were present in the culture medium used as the enzyme preparation, it was assumed that recombinant chitinase from *T. lanuginosus* shows activity against colloidal chitin, while recombinant chitosanase from *A. fumigatus* was active only against chitosan dissolved in 2% (v/v) acetic acid. However, it should be remembered that acetylated (GlcNAc) and deacetylated (GlcN) residues occur in both chitin and chitosan and can be hydrolysed by both enzymes. Determined in this way, the specific activity of the recombinant chitinase was 2.9 U mg^−1^. Determined based on densitometric analysis of the SDS gel (Figure 6; Supplementary Figure S4), the concentration of recombinant chitinase was 63 μg ml^−1^, which allowed to determine the adjusted specific activity of 51.03 U mg^−1^. Recombinant chitinase from *T. lanuginosus* expressed in *P. pastoris* GS115 in addition to activity against synthetic substrates such as: 4-nitrophenyl N,N′-diacetyl-β-D-chitobioside (exochitinolytic, 30 mU mg^−1^) and 4-nitrophenyl β-D-N,N′,N”- triacetylchitotriose (endochitinolytic, 150 mU mg^−1^), also showed activity against colloidal chitin. However, this value was not disclosed (Zhang et al., 2015). Chitinase from *Bacillus licheniformis* expressed in *P. pastoris* KM71H yeast showed a specific activity of 3.4 mU mg^−1^ protein in the culture medium (Menghiu et al., 2019). On the other hand, chitinase from *Streptomyces albolongus* ATCC 27414 expressed in *E. coli* BL21 bacteria showed specific activity against colloidal chitin of as much as 66.2 Umg^−1^of protein (Guo et al., 2019). This indicates the wide range of activities that biocatalysts from various sources exhibit.

The activity of the third enzyme expressed in the polycistronic system pCHIT was 5.2 U mg^−1^ against 0.5% (w/v) of chitosan dissolved in acetic acid. After taking into account the concentration of recombinant chitosanase in the after-culture medium (determined on the basis of the intensity of the band), the corrected specific activity was 11.73 U mg^-^1 (Table 3; Supplementary Figure S4). Previous studies focused on the expression of this enzyme in the yeast *P. pastoris* GS115 at the bioreactor scale of (Chen et al., 2012). The pre-purified recombinant chitosanase obtained from *A. fumigatus* showed a specific activity of 8.3 U mg^−1^. The higher activity of the enzyme obtained in our research may be due to the fact that recombinant chitinase hydrolysing glycosidic bonds on acetylated units was also present in the reaction mixture. The chitosan used as a substrate for the determination of chitosanase activity showed a deacetylation degree of 78.13 ± 0.14%. This indicates that acetylated units susceptible to the action of recombinant chitinase were present in the substrate. The ability of the multi-enzyme cocktail to modify colloidal chitin was confirmed by GPC/SEC analysis of products obtained as a result of the action of recombinant proteins present in the after-culture medium (Supplementary Figure S5). The number-average (Mn) and weight-average (Mw) molar masses of the substrate and the obtained products determined on the basis of the obtained results clearly indicate the occurrence of the enzymatic depolymerization of colloidal chitin (Supplementary Table S4). Meanwhile the polydispersity index ∼1.0 indicates that there is a high degree of homogeneity among these degradation products suggesting that chitooligosaccharides of similar chain length have been synthesized. Unfortunately, the set of columns used in the analysis did not allow to accurately determine the masses of the products obtained during enzymatic degradation of the biopolymer.

To the best of our knowledge, our research is the first attempt at a comprehensive, multi-enzymatic modification of chitin substrates using a mixture of several enzymes belonging to different classes and produced by one host. Chyleński et al. used Design of Experiment (DoE) approach to create highly efficient enzyme cocktails for the complete enzymatic hydrolysis of lignocellulosic biomass (Chylenski et al., 2017). Two-stage enzymatic hydrolysis was carried out using five different enzyme mixture components. The chitin-rich biomass was also subjected to a total saccharification process using enzymatic cocktail. The DoE strategy included the use of five mono-component enzymes from *Serratia marcescens*, three chitinases, SmChiA, SmChiB, SmChiC, a lytic polysaccharide monooxygenase, SmLPMO10A, and a β-N-acetylhexosaminidase (SmCHB). The enzymes were heterologously expressed in monogenic *E. coli* expression systems. The optimised enzyme cocktail resulted in the saccharification degree of shrimp and crab chitins of 70–75%. Meanwhile, a “minimal” cocktail yielded only 40% saccharification (Mekasha et al., 2017). The increase of yield affirms the hypothesis that chitinolytic enzymes interact synergistically with each other in processing chitin and its derivatives. Of course, the DoE approach assumes a wider optimisation of the reaction conditions (enzymes, mix ratios, temperature, pH, salt, and cofactors), but it does not assume the simultaneous production of biocatalysts in one host. Therefore, the costs of the processes are multiplied by the necessity to produce and purify recombinant enzymes that are involved in multi-enzyme cocktails.

Our research has shown that the use of polycistronic expression of chitin- and chitosanolytic enzymes using self-processing A2 sequences has a real chance to overcome these limitations. Moreover, the use of efficient *P. pastoris* expression systems that produce small amounts of native extracellular proteins allows the application of raw culture medium as enzyme preparations significantly facilitating the recombinant proteins purification. However, careful examinations of the relationships between the efficiency of the expression of individual genes, their location in the multi-gene expression cassette, gene properties (size, spatial protein structure) and helper sequences (secretory sequences, A2 sequences) are still necessary.

## Conclusion

In this work we have expressed and characterised two chitin deacetylases from *M. circinelloides* IBT-83 and utilised one of these proteins for the synthesis of the first eukaryotic, polycistronic expression system employing self-processing 2A sequences containing three sequentially acting chitin processing enzymes. During the study it was shown that using viral self-processing sequences and degeneracy of the genetic code it is possible to stably express synergistically acting extracellular biocatalysts under control of the same promoter. These findings will hopefully translate into more effective conversion of chitin waste into their valuable derivatives: chitosan and chitooligosaccharides.

## Data Availability

The original contributions presented in the study are included in the article/Supplementary Materials, further inquiries can be directed to the corresponding author.

## References

[B1] Al Sagheer F. A. Al-Sughayer M. A. Muslim S. Elsabee M. Z. (2009). Extraction and Characterization of Chitin and Chitosan from marine Sources in Arabian Gulf. Carbohydr. Polym. 77, 410–419. 10.1016/j.carbpol.2009.01.032

[B2] Andrés E. Albesa-Jové D. Biarnés X. Moerschbacher B. M. Guerin M. E. Planas A. (2014). Structural Basis of Chitin Oligosaccharide Deacetylation. Angew. Chem. Int. Ed. 53, 6882–6887. 10.1002/anie.201400220 24810719

[B3] Blair D. E. Hekmat O. Schüttelkopf A. W. Shrestha B. Tokuyasu K. Withers S. G. (2006). Structure and Mechanism of Chitin Deacetylase from the Fungal Pathogen *Colletotrichum Lindemuthianum*†,‡. Biochemistry 45, 9416–9426. 10.1021/bi0606694 16878976

[B4] Chatterjee S. Adhya M. Guha A. K. Chatterjee B. P. (2005). Chitosan from Mucor Rouxii: Production and Physico-Chemical Characterization. Process Biochem. 40, 395–400. 10.1016/j.procbio.2004.01.025

[B5] Chen X. Zhai C. Kang L. Li C. Yan H. Zhou Y. (2012). High-level Expression and Characterization of a Highly Thermostable Chitosanase from *Aspergillus fumigatus* in Pichia pastoris. Biotechnol. Lett. 34, 689–694. 10.1007/s10529-011-0816-0 22160328

[B6] Chylenski P. Forsberg Z. Ståhlberg J. Várnai A. Lersch M. Bengtsson O. (2017). Development of Minimal Enzyme Cocktails for Hydrolysis of Sulfite-Pulped Lignocellulosic Biomass. J. Biotechnol. 246, 16–23. 10.1016/j.jbiotec.2017.02.009 28219736

[B7] De Felipe P. Luke G. A. Hughes L. E. Gani D. Halpin C. Ryan M. D. (2006). E Unum Pluribus: Multiple Proteins from a Self-Processing Polyprotein. Trends Biotechnol. 24, 68–75. 10.1016/j.tibtech.2005.12.006 16380176

[B21] De Kok S. d. Stanton L. H. Slaby T. Durot M. Holmes V. F. Patel K. G. (2014). Rapid and Reliable DNA Assembly via Ligase Cycling Reaction. ACS Synth. Biol. 3, 97–106. 10.1021/sb4001992 24932563

[B8] Donnelly M. L. L. Hughes L. E. Luke G. Mendoza H. Ten Dam E. Gani D. (2001a). The 'cleavage' Activities of Foot-And-Mouth Disease Virus 2A Site-Directed Mutants and Naturally Occurring '2A-like' Sequences. J. Gen. Virol. 82, 1027–1041. 10.1099/0022-1317-82-5-1027 11297677

[B9] Donnelly M. L. L. Luke G. Mehrotra A. Li X. Hughes L. E. Gani D. (2001b). Analysis of the Aphthovirus 2A/2B Polyprotein 'cleavage' Mechanism Indicates Not a Proteolytic Reaction, but a Novel Translational Effect: A Putative Ribosomal 'skip'. J. Gen. Virol. 82, 1013–1025. 10.1099/0022-1317-82-5-1013 11297676

[B10] FAO (2017). Increased Production of Farmed Shrimp Leads to Improved International Trade. Food and Agriculture organizatioin of the United Nations. Available at: http://www.fao.org/in-action/globefish/market-reports/resource-detail/en/c/989543/(Accessed September 4, 2020).

[B11] Gauthier C. Clerisse F. Dommes J. Jaspar-Versali M.-F. (2008). Characterization and Cloning of Chitin Deacetylases From Rhizopus Circinans. Protein Expr. Purif. 59, 127–137. 10.1016/j.pep.2008.01.013 18314348

[B12] Geier M. Fauland P. Vogl T. Glieder A. (2015). Compact Multi-Enzyme Pathways in P. Pastoris. Chem. Commun. 51, 1643–1646. 10.1039/c4cc08502g 25502218

[B13] Ghormade V. Pathan E. K. Deshpande M. V. (2017). Can Fungi Compete With marine Sources for Chitosan Production?. Int. J. Biol. Macromol. 104, 1415–1421. 10.1016/j.ijbiomac.2017.01.112 28143744

[B14] Grifoll-Romero L. Pascual S. Aragunde H. Biarnés X. Planas A. (2018). Chitin Deacetylases: Structures, Specificities, and Biotech Applications. Polymers (Basel) 10, 1–29. 10.3390/polym10040352 PMC6415152 30966387

[B15] Guo N. Sun J. Wang W. Gao L. Liu J. Liu Z. (2019). Cloning, Expression and Characterization of a Novel Chitosanase from Streptomyces Albolongus ATCC 27414. Food Chem. 286, 696–702. 10.1016/j.foodchem.2019.02.056 30827665

[B16] Halpin C. Cooke S. E. Barakate A. Amrani A. E. Ryan M. D. (1999). Self-processing 2A-Polyproteins - a System for Co-ordinate Expression of Multiple Proteins in Transgenic Plants. Plant J. 17, 453–459. 10.1046/j.1365-313X.1999.00394.x 10205902

[B17] Hayes M. (2012). “Chitin, Chitosan and Their Derivatives from marine Rest Raw Materials: Potential Food and Pharmaceutical Applications,” In Marine Bioactive Compounds: Sources, Characterization And Applications. Boston, MA: Springer, 1–229. 10.1007/978-1-4614-1247-2

[B18] Kaczmarek M. Struszczyk-Świta K. Struszczyk-Świta K. Florczak T. Szczęsna-Antczak M. Antczak T. (2016). Isolation, Molecular Cloning and Characterisation of Two Genes Coding Chitin Deacetylase from Mucor Circinelloides IBT-83. Pcacd 21, 93–103. 10.15259/PCACD.21.09

[B19] Kaczmarek M. B. Struszczyk-swita K. Li X. Szczęsna-Antczak M. Daroch M. (2019). Enzymatic Modifications of Chitin, Chitosan, and Chitooligosaccharides. Front. Bioeng. Biotechnol. 7, 243. 10.3389/fbioe.2019.00243 31612131 PMC6776590

[B20] Kjeldsen T. (2000). Yeast Secretory Expression of Insulin Precursors. Appl. Microbiol. Biotechnol. 54, 277–286. 10.1007/s002530000402 11030562

[B22] Kolodziejska I. Malesa-cieçwierz M. Lerska A. Sikorski Z. (1999). Properties of Chitin Deacetylase from Crude Extracts of Mucor Rouxii Mycelium. J. Food Biochem. 23, 45–57. 10.1111/j.1745-4514.1999.tb00004.x

[B23] Liang Y. Tang J. Luo Y. Kaczmarek M. B. Li X. Daroch M. (2019). Thermosynechococcus as a Thermophilic Photosynthetic Microbial Cell Factory for CO2 Utilisation. Bioresour. Tech. 278, 255–265. 10.1016/j.biortech.2019.01.089 30708328

[B24] Liu P. Sehaqui H. Tingaut P. Wichser A. Oksman K. Mathew A. P. (2014). Cellulose and Chitin Nanomaterials for Capturing Silver Ions (Ag+) from Water via Surface Adsorption. Cellulose 21, 449–461. 10.1007/s10570-013-0139-5

[B25] Marei N. H. El-Samie E. A. Salah T. Saad G. R. Elwahy A. H. M. (2016). Isolation and Characterization of Chitosan from Different Local Insects in Egypt. Int. J. Biol. Macromol. 82, 871–877. 10.1016/j.ijbiomac.2015.10.024 26459168

[B26] Martinou A. Kafetzopoulos D. Bouriotis V. (1993). Isolation of Chitin Deacetylase from Mucor Rouxii by Immunoaffinity Chromatography. J. Chromatogr. A 644, 35–41. 10.1016/0021-9673(93)80117-Q

[B27] Mekasha S. Byman I. R. Lynch C. Toupalová H. Anděra L. Næs T. (2017). Development of Enzyme Cocktails for Complete Saccharification of Chitin Using Mono-Component Enzymes from *Serratia marcescens* . Process Biochem. 56, 132–138. 10.1016/j.procbio.2017.02.021

[B28] Menghiu G. Ostafe V. Prodanovic R. Fischer R. Ostafe R. (2019). Biochemical Characterization of Chitinase A from Bacillus Licheniformis DSM8785 Expressed in Pichia pastoris KM71H. Protein Expr. Purif. 154, 25–32. 10.1016/j.pep.2018.09.007 30237128

[B29] Muzzarelli R. A. A. Rocchetti R. Stanic V. Weckx M. (1997). Methods for the Determination of the Degree of Acetylation of Chitin and Chitosan. Chitin Handb, 109–119.

[B30] Nakamura A. M. Nascimento A. S. Polikarpov I. (2017). Structural Diversity of Carbohydrate Esterases. Biotechnol. Res. Innovation 1, 35–51. 10.1016/j.biori.2017.02.001

[B31] Nelson N. (1944). A Photometric Adaptation of the Somogyi for the Determination of Glucose. J. Biol. Chem. 153, 375–381. 10.1016/S0021-9258(18)71980-7

[B32] Nguyen S. T. C. Freund H. L. Kasanjian J. Berlemont R. (2018). Function, Distribution, and Annotation of Characterized Cellulases, Xylanases, and Chitinases from CAZy. Appl. Microbiol. Biotechnol. 102, 1629–1637. 10.1007/s00253-018-8778-y 29359269 PMC5806127

[B33] Pareek N. Vivekanand V. Agarwal P. Saroj S. Singh R. P. (2013). Bioconversion to Chitosan: A Two Stage Process Employing Chitin Deacetylase from Penicillium oxalicum SAEM-51. Carbohydr. Polym. 96, 417–425. 10.1016/j.carbpol.2013.04.005 23768582

[B34] Rath A. Glibowicka M. Nadeau V. G. Chen G. Deber C. M. (2009). Detergent Binding Explains Anomalous SDS-PAGE Migration of Membrane Proteins. Pnas 106, 1760–1765. 10.1073/pnas.0813167106 19181854 PMC2644111

[B35] Roth T. L. Milenkovic L. Scott M. P. (2014). A Rapid and Simple Method for DNA Engineering Using Cycled Ligation Assembly. PLoS One 9, e107329–9. 10.1371/journal.pone.0107329 25226397 PMC4167330

[B36] Sievers F. Higgins D. G. (2018). Clustal Omega for Making Accurate Alignments of many Protein Sequences. Protein Sci. 27, 135–145. 10.1002/pro.3290 28884485 PMC5734385

[B37] Sinha J. Plantz B. A. Inan M. Meagher M. M. (2005). Causes of Proteolytic Degradation of Secreted Recombinant Proteins Produced in Methylotrophic yeastPichia Pastoris: Case Study with Recombinant Ovine Interferon-?. Biotechnol. Bioeng. 89, 102–112. 10.1002/bit.20318 15580575

[B38] Souza C. P. Burbano-Rosero E. M. Almeida B. C. Martins G. G. Albertini L. S. Rivera I. N. G. (2009). Culture Medium for Isolating Chitinolytic Bacteria from Seawater and Plankton. World J. Microbiol. Biotechnol. 25, 2079–2082. 10.1007/s11274-009-0098-z

[B39] Struszczyk M. H. (2000). Herstellung von Chitosan und einige Anwendungen. Available at: https://www.researchgate.net/publication/252779927_Herstellung_von_Chitosan_und_einige_Anwendungen (Accessed January 24, 2017).

[B40] Suryawanshi N. Jujjavarapu S. E. Ayothiraman S. (2019). Marine Shell Industrial Wastes-An Abundant Source of Chitin and its Derivatives: Constituents, Pretreatment, Fermentation, and Pleiotropic Applications-A Revisit. Int. J. Environ. Sci. Technol. 16, 3877–3898. 10.1007/s13762-018-02204-3

[B41] Synowiecki J. Al-Khateeb N. A. A. Q. (1997). Mycelia of Mucor Rouxii as a Source of Chitin and Chitosan. Food Chem. 60, 605–610. 10.1016/S0308-8146(97)00039-3

[B42] Synowiecki J. Al-Khateeb N. A. (2003). Production, Properties, and Some New Applications of Chitin and its Derivatives. Crit. Rev. Food Sci. Nutr. 43, 145–171. 10.1080/10408690390826473 12705640

[B43] Szczesna-Antczak M. Antczak T. Piotrowicz-Wasiak M. Rzyska M. Binkowska N. Bielecki S. (2006). Relationships between Lipases and Lipids in Mycelia of Two Mucor Strains. Enzyme Microb. Technol. 39, 1214–1222. 10.1016/j.enzmictec.2006.03.008

[B44] Thiago R. d. S. M. Pedro P. M. d. M. Eliana F. C. S. (2014). Solid Wastes in Brewing Process: A Review. J. Brew. Distill. 5, 1–9. 10.5897/jbd2014.0043

[B45] Tokuyasu K. Ohnishi-Kameyama M. Hayashi K. (1996). Purification and Characterization of Extracellular Chitin Deacetylase fromColletotrichum Lindemuthianum. Biosci. Biotechnol. Biochem. 60, 1598–1603. 10.1271/bbb.60.1598 8987657

[B46] Tuveng T. R. Rothweiler U. Udatha G. Vaaje-Kolstad G. Smalås A. Eijsink V. G. H. (2017). Structure and Function of a CE4 Deacetylase Isolated from a marine Environment. PLoS One 12, e0187544–15. 10.1371/journal.pone.0187544 29107991 PMC5673215

[B47] Zhang M. Puri A. K. Govender A. Wang Z. Singh S. Permaul K. (2015). The Multi-Chitinolytic Enzyme System of the Compost-Dwelling Thermophilic Fungus Thermomyces Lanuginosus. Process Biochem. 50, 237–244. 10.1016/j.procbio.2014.11.008

[B48] Zhang S. Chen Z. Wen Q. Yang L. Wang W. Zheng J. (2016). Effectiveness of Bulking Agents for Co-composting Penicillin Mycelial Dreg (PMD) and Sewage Sludge in Pilot-Scale System. Environ. Sci. Pollut. Res. 23, 1362–1370. 10.1007/s11356-015-5357-y 26362639

[B49] Zhao Y. Jo G.-H. Ju W.-T. Jung W.-J. Park R.-D. (2011). A HighlyN-Glycosylated Chitin Deacetylase Derived from a Novel Strain ofMortierellasp. DY-52. Biosci. Biotechnol. Biochem. 75, 960–965. 10.1271/bbb.110011 21597184

[B50] Zhu X.-Y. Zhao Y. Zhang H.-D. Wang W.-X. Cong H.-H. Yin H. (2019). Characterization of the Specific Mode of Action of a Chitin Deacetylase and Separation of the Partially Acetylated Chitosan Oligosaccharides. Mar. Drugs 17, 74–15. 10.3390/md17020074 PMC6409515 30678277

