# Supplementary material for: Polycistronic Expression System for Pichia pastoris Composed of Chitino- and Chitosanolytic Enzymes
Source: Front Bioeng Biotechnol. 2021 Aug 18;9:710922. doi: 10.3389/fbioe.2021.710922 (PMC8418187; doi:10.3389/fbioe.2021.710922)
Supplement: Supplementary file 1 [file DataSheet1.docx]

Polycistronic expression system for *Pichia pastoris* composed of chitino- and chitosanolytic enzymes

Michal B. Kaczmarek ^1,2^, Katarzyna Struszczyk-Swita^,2^, Meng Xiao ^1^, Mirosława Szczęsna-Antczak ^2^, Tadeusz Antczak ^2^ , Magdalena Gierszewska ^3^, Alexander Steinbüchel ^4^, Maurycy Daroch ^1^*

^1^ School of Environment and Energy, Peking University Shenzhen Graduate School, Shenzhen, 518055, China

^2^ Institute of Molecular and Industrial Biotechnology, Lodz University of Technology, Stefanowskiego 4/40, 90-924 Lodz, Poland

^3^ Department of Physical Chemistry and Physicochemistry of Polymers, Faculty of Chemistry, Nicolaus Copernicus University in Toruń, Gagarina 7, 87-100 Toruń, Poland

^4^ International Center for Research on Innovative Biobased Materials (ICRI-BioM) – International Research Agenda, Lodz University of Technology, Żeromskiego 116, 90-924 Lodz, Poland

*** Correspondence:**Maurycy Daroch
m.daroch@pkusz.edu.cn

Keywords: chitin, chitosan, enzymatic modification, polycistronic expression, self-processing 2A sequence, chitin deacetylase, chitinase, chitosanase

SUPPLEMENTARY MATERIALS

Supplementary Table 1. DNA fragments used for the synthesis of the pCHIT vector and primers used for their amplification.

| No. | pCHIT element | Primer | Sequence | Size [bp] |
| --- | --- | --- | --- | --- |
| I | *chda*II | chda_LCRF | 5’**P-**GCTACTTCCACCAAATCCGCC-3’ | 1299 |
|  |  | chda_LCRR | 5’**P**-GAAAATGTAAGCAGCAACGGCA-3’ |  |
| II | P2A | P2A_LCRF | 5’**P-**GCTACTAACTTTTCCTTGTTGAA-3’ | 57 |
|  |  | P2A_LCRR | 5’**P**-TGGACCTGGATTCTCTTCGA-3’ |  |
| III | α-factorII *+chit* | chit_LCRF | 5’**P-**ATGAGGTTTCCATCAATATTCACCG-3’ | 1467 |
|  |  | chit_LCRR | 5’**P**-TTCACCTGGGAAACCGGCAC-3’ |  |
| IV | T2A | T2A_LCRF | 5’**P**-GAAGGTAGAGGTTCTTTGTTAAC-3’ | 54 |
|  |  | T2A_LCRR | 5’**P**-TGGTCCAGGGTTTTCCTC-3’ |  |
| V | α-factorIII *+chto* | chto_LCRF | 5’**P-** ATGAGATTCCCTTCCATCTTCACC-3’ | 933 |
|  |  | chto_LCRR | 5’**P**-TTAAGCCTTCAAACCGGCAACC-3’ |  |
| VI | pPICZ | vector_LCRF | 5’**P-** GTTTGTAGCCTTAGACATGACTGTTCC -3’ | 3470 |
|  |  | vector_LCRR | 5’**P**-TGCTTCAGCCTCTCTCTTCTCGAGA-3’ |  |

Supplementary Table 2. Bridging oligonuleotides used for the synthesis of the pCHIT vector using LCR assembly method.

| No. | Bridging oligonucleotide | Sequence |
| --- | --- | --- |
| 1 | pPICZ_chdaII | 5’-GTATCTCTCGAGAAGAGAGAGGCTGAAGCA GCTACTTCCACCAAATCCGCC-3’ |
| 2 | chdaII_P2A | 5’-TGCCGTTGCTGCTTACATTTTC GCTACTAACTTTTCCTTGTTGAAGC-3’ |
| 3 | P2A_chit | 5’-GATGTCGAAGAGAATCCAGGTCCA ATGAGGTTTCCATCAATATTCACCGC-3’ |
| 4 | chit_T2A | 5’-GTGCCGGTTTCCCAGGTGAA GAAGGTAGAGGTTCTTTGTTAACTTGT-3’ |
| 5 | T2A_chto | 5’-CGTTGAGGAAAACCCTGGACCA ATGAGATTCCCTTCCATCTTCACC-3’ |
| 6 | chto_pPICZ | 5’-GGTTGCCGGTTTGAAGGCTTAA GTTTGTAGCCTTAGACATGACTGTTC-3’ |

Supplementary Table 3. Primers used for colony PCR analysis of *P. pastoris* KM71H_pCHIT transformants.

| Reaction | Primer | annealing  temp. | Sequence |
| --- | --- | --- | --- |
| 1 | chdaII_cF | 54°C | 5’**-**GCTACTTCCACCAAATCCGCC-3’ |
|  | chdaII_cR |  | 5’-GAAAATGTAAGCAGCAACGGCA-3’ |
| 2 | chit_cF | 54°C | 5’**-**ATGGCTACCGGATTCAGAACT-3’ |
|  | chit_cR |  | 5’-TTCACCTGGGAAACCGGCAC-3’ |
| 3 | chto_cF | 54°C | 5’**-**TTACAACTTGCCAAACAACTTGAAGC-3’ |
|  | chto_cR |  | 5’-TTAAGCCTTCAAACCGGCAACC-3’ |
| 4 | check_1 | 54°C | 5’-TGCCGTTGCTGCTTACATTTTC-3’ |
|  | chit_cR |  | 5’-TTCACCTGGGAAACCGGCAC-3’ |
| 5 | check_2 | 57°C | 5’-AGAGTCCAAGTATGACAATTTGCGT-3’ |
|  | chto_cR |  | 5’-TTAAGCCTTCAAACCGGCAACC-3’ |

Supplementary Table 4. Summary of number-average molar masses (Mn) weight-average molar masses (Mw) and polydispersion coefficients (Mw/Mn) determined during GPC/SEC analysis of products obtained as a result of enzymatic degradation of colloidal chitin with the use of recombinant proteins. The after culture medium obtained after the methanol-induced cultivation of the transformant *Pichia pastoris* KM71H_pCHIT was used as the enzyme preparation.

|  | Control sample | | | |
| --- | --- | --- | --- | --- |
|  | Peak 1 | U* | Peak 2 | U* |
| Mn [g/mol] | 2 323 | 0,9 | - | - |
| Mw [g/mol] | 2 397 | 0,5 | - | - |
| Polydispersity factor  Mw/Mn | 1.03 | 1.0 | - | - |
|  | **Sample 1** | | | |
| Mn [g/mol] | 2 153 | 0.5 | 1 570 | 1.7 |
| Mw [g/mol] | 2 193 | 0.5 | 1 577 | 1.5 |
| Polydispersity factor  Mw/Mn | 1.02 | 0 | 1,00 | 0.6 |
|  | **Sample 2** | | | |
| Mn [g/mol] | 2 160 | 0.5 | 1 573 | 0.7 |
| Mw [g/mol] | 2 207 | 0,5 | 1 573 | 1,0 |
| Polydispersity factor  Mw/Mn | 1,02 | 0,6 | 1,01 | 0,6 |

* standard deviation

| 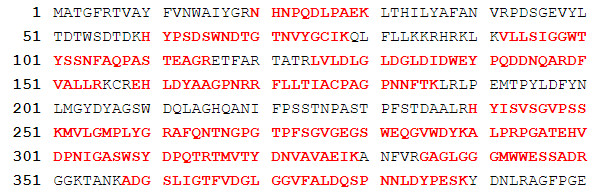 | |
| --- | --- |
| Protein ID:  Database:  Score:  Nominal mass (M_r_):  Calculated pI:  Taxonomy:  Protein sequence coverage: | AIW06013.1  NCBIprot  50926  44280  5.66  *Thermomyces lanuginosus*  62% |

Supplementary Figure 1. Protein identification results by LC-MS/MS obtained in MASCOT. Identification of chitinase from *Thermomyces lanuginosus*; Score - scoring -10 * Log (P), where P is the probability that the observed match is a random event; Mr - reference protein mass; Calculated pI - theoretical value of the isoelectric point of the reference protein; Taxonomy - reference protein taxonomy; the red color marks the amino acid sequences of the peptides for which sequence homology with the sequence of the reference protein was observed.

| 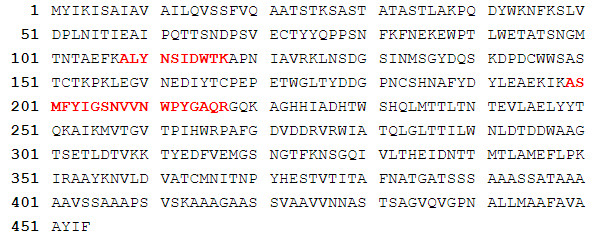 | |
| --- | --- |
| Protein ID:  Database:  Score:  Nominal mass (M_r_):  Calculated pI:  Taxonomy:  Protein sequence coverage: | OAD01792.1  NCBIprot  488  49416  5.07  *Mucor circinelloides* f. *lusitanicus* CBS 277.49  6% |

Supplementary Figure 2. Protein identification results by LC-MS/MS obtained in MASCOT. Identification of chitin deacetylase from *Mucor circinelloides* IBT-83; Score - scoring -10 * Log (P), where P is the probability that the observed match is a random event; Mr - reference protein mass; Calculated pI - theoretical value of the isoelectric point of the reference protein; Taxonomy - reference protein taxonomy; the red color marks the amino acid sequences of the peptides for which sequence homology with the sequence of the reference protein was observed.

| 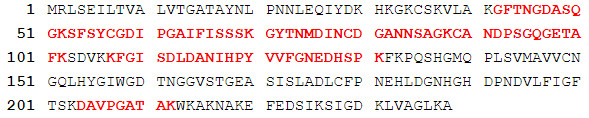 | |
| --- | --- |
| Protein ID:  Database:  Score:  Nominal mass (M_r_):  Calculated pI:  Taxonomy:  Protein sequence coverage: | ABZ88800.1  NCBIprot  17167  25517  6.04  *Aspergillus* sp. CJ22-326  39% |

Supplementary Figure 3. Protein identification results by LC-MS/MS obtained in MASCOT. Identification of chitosanase from *Aspergillus fumigatus*; Score - scoring -10 * Log (P), where P is the probability that the observed match is a random event; Mr - reference protein mass; Calculated pI - theoretical value of the isoelectric point of the reference protein; Taxonomy - reference protein taxonomy; the red color marks the amino acid sequences of the peptides for which sequence homology with the sequence of the reference protein was observed.


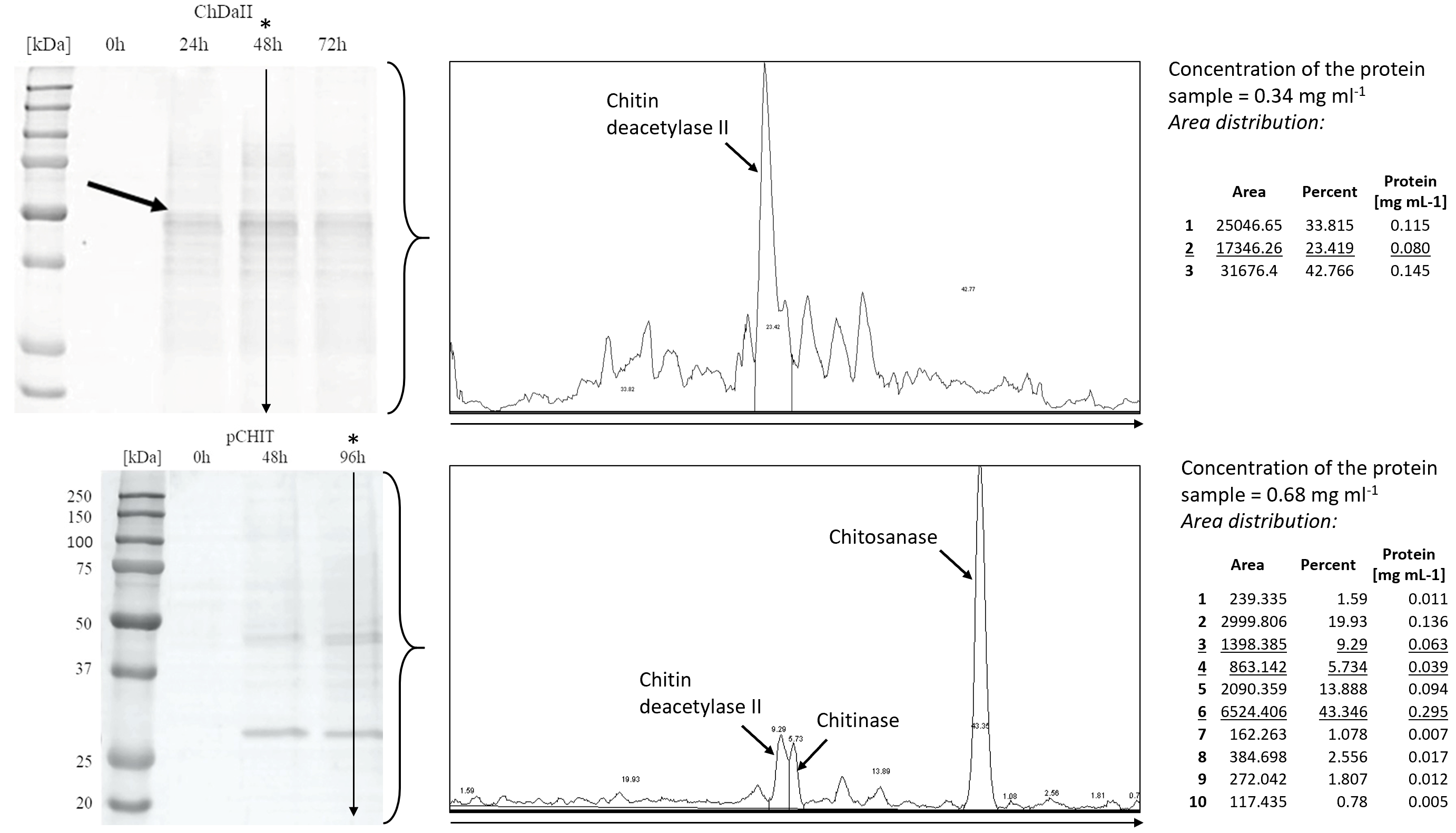


**Supplementary Figure 4.** Densitometric analysis of Figure 3 (top) and Figure 6 (bottom) using ImageJ software (<https://imagej.nih.gov/ij/index.html>). Gels were converted into chromatograms. After setting the baselines the chromatograms have been divided into areas that correspond to the migration of different proteins in during electrophoresis. Areas corresponding to the proteins constituting the construct have been labeled on the figure and their area was used for the calculation of the adjusted enzymatic activities.


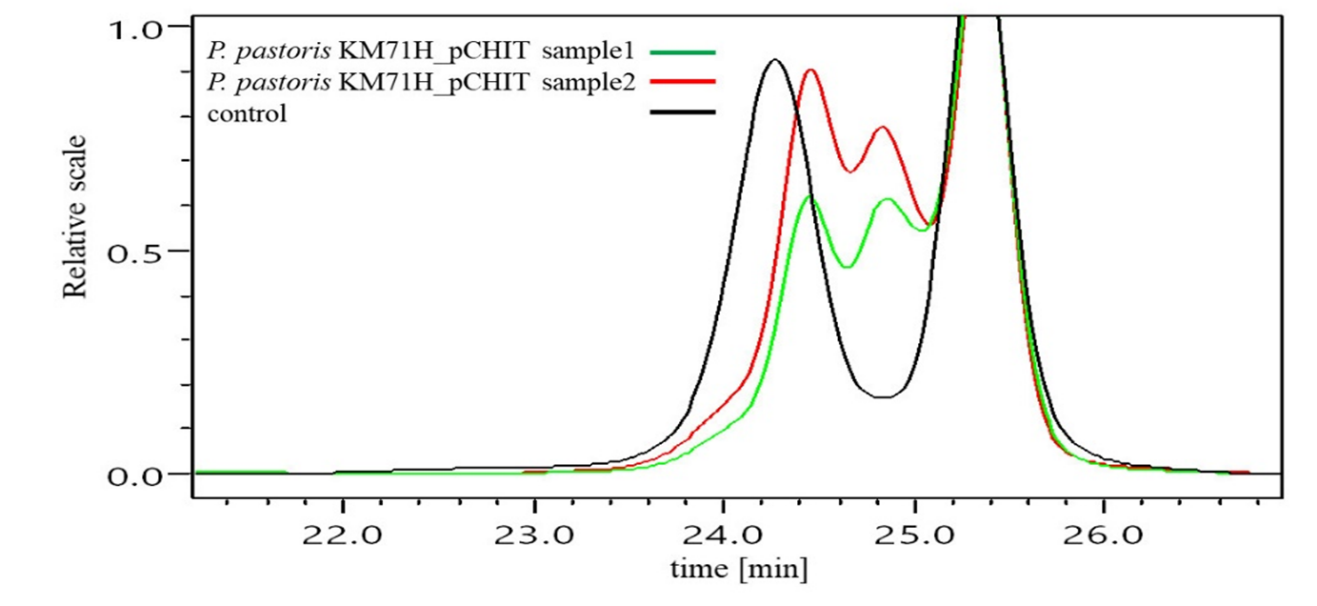
Supplementary Figure 5. GPC/SEC analysis of products obtained by enzymatic degradation of colloidal chitin with the use of recombinant proteins. The culture medium obtained after the methanol-induced cultivation of the transformant *Pichia pastoris* KM71H_pCHIT was used as the enzyme preparation.
